# Efficiency of a phone coaching program on adherence to continuous positive airway pressure in sleep apnea hypopnea syndrome: a randomized trial

**DOI:** 10.1186/s12890-015-0099-7

**Published:** 2015-09-14

**Authors:** Kamila Sedkaoui, Ludivine Leseux, Sandrine Pontier, Nicole Rossin, Paul Leophonte, Jean-Louis Fraysse, Alain Didier

**Affiliations:** Service de pneumologie – allergologie, hôpital Larrey, CHU de Toulouse, chemin de Pouvourville, 31059 Toulouse, France; SADIR association, oncopole, 2 place Pierre Potier, CS 40623, 31106 Toulouse cedex 1, France; SADIR assistance, oncopole, 2 place Pierre Potier, CS 40623, 31106 Toulouse cedex 1, France

## Abstract

**Background:**

Continuous Positive Airway Pressure (CPAP) remains the reference treatment for moderate to severe forms of the Sleep Apnea/Hypopnea Syndrome (SAHS). Compliance to the treatment appears to be a key factor to improving health status of these patients.

**Methods:**

We conducted a multicenter, prospective, randomized, controlled, parallel group trial of standard support completed or not within 3 months of coaching sessions for newly diagnosed SAHS patients starting CPAP therapy. This study has been recorded by AFSSAPS with the RCB number: 2009-A01127-50 and received favourably by the Human Studies Committee in France. The coaching session consisted of 5 sessions of telephone-based counselling by competent staff. The primary outcome was the proportion of patients using CPAP more than 3 h per night for 4 months; the secondary outcome was mean hours of CPAP usage in the 2 groups.

**Results:**

Three hundred and seventy-nine patients fulfilled the inclusion criteria and were randomized. The percentage of patients using CPAP more than 3 h per night for 4 months was 65 % for the standard support group and 75 % for the coached group. This difference reached a statistical significance (χ2 = 3.97). The mean CPAP usage was increased in the coached group versus standard group. A difference of 26 min was observed (4 h34+/−2 h17 and 4 h08+/−2 h25 respectively, *p* = 0.04).

**Conclusion:**

This study shows that SAHS patients who benefit from phone coaching are statistically more compliant to CPAP than a standard support group is. A simple phone coaching procedure based on knowledge of the disease and reinforcement messages about treatment benefits helps to improve CPAP adherence in SAHS patients.

**Trial registration:**

NCT02435355

## Background

Sleep Apnea/Hypopnea Syndrome (SAHS) is a sleep disorder, which affects about 4 % of the general population, causing excessive daytime sleepiness. SAHS is associated with increased risk of road accidents [1] and high level of co-morbidities such as arterial hypertension (AHT) or metabolic disorder [2–5]. Continuous Positive Airway Pressure (CPAP) remains the reference treatment for moderate to severe forms of the disease [6, 7]. It is at present well established that symptoms of SAHS are proportionally correlated with the duration of use of CPAP (compliance). SAHS patients who used CPAP for less than an hour a day have significantly lower survival rates compared to patients who have high (>6 h/d) or even moderate (1 to 6 h/d) compliance [8]. Weaver et al. demonstrated a linear dose–response relationship between increased CPAP use and achieving normal levels for objective and subjective daytime sleepiness [9]. CPAP treatment also reduces co-morbid conditions. For example in a patient with AHT the most significant reduction in blood pressure was observed in patients who used CPAP for more than 5.6 h per night [10].

Compliance to CPAP treatment appears to be a key factor in improving the health status of patients with moderate to severe SAHS. CPAP non-adherence is a significant barrier to SAHS treatment. However, the first six-month period appears critical for long term adherence [11–13]. Refusal rate for adherence is ranges from 5 to 50 % in the first week to 6 months [14, 15]. The percentage of patients’ adherence to CPAP after 5 and 10 years was 81 and 70 % respectively [16]. The severity of the disease and the presence of daytime sleepiness are strong predictive factors for subsequent adherence [17]. Other factors, such as sex, age, socio-economic status and personality traits are less robust predictors [17]. In order to improve CPAP adhesion, several support and patient education programs have been developed, ranging from a weekly phone call to the development of an intensive patient education program [18–20]. All these studies showed an enhancement of the use of CPAP, but the number of patients included was often small. Only two randomized trials have involved more than 40 patients in each arm [21, 22].

The French system of CPAP equipment has some specificity. After the diagnosis has been confirmed and medical prescription has been performed, a home care provider makes CPAP available to patients at home. He equips the patients with a mask and device and collects CPAP data registered by the machine. These data are sent to physicians after 1 month and 4 months then every 6 months. CPAP equipment is reimbursed by the national health insurance if its daily use is superior to 3 h per night for the first five months. If this condition is met, health insurance coverage is maintained and compliance is checked every year. If this condition is not respected, the national health system might decide to stop reimbursement.

The purpose of the present study was to determine whether a coaching session based on phone calls improved compliance with CPAP in participants with SAHS in a randomized trial. We have evaluated and validated feasibility of this coaching session [23].

## Methods

**Fig. 1:**
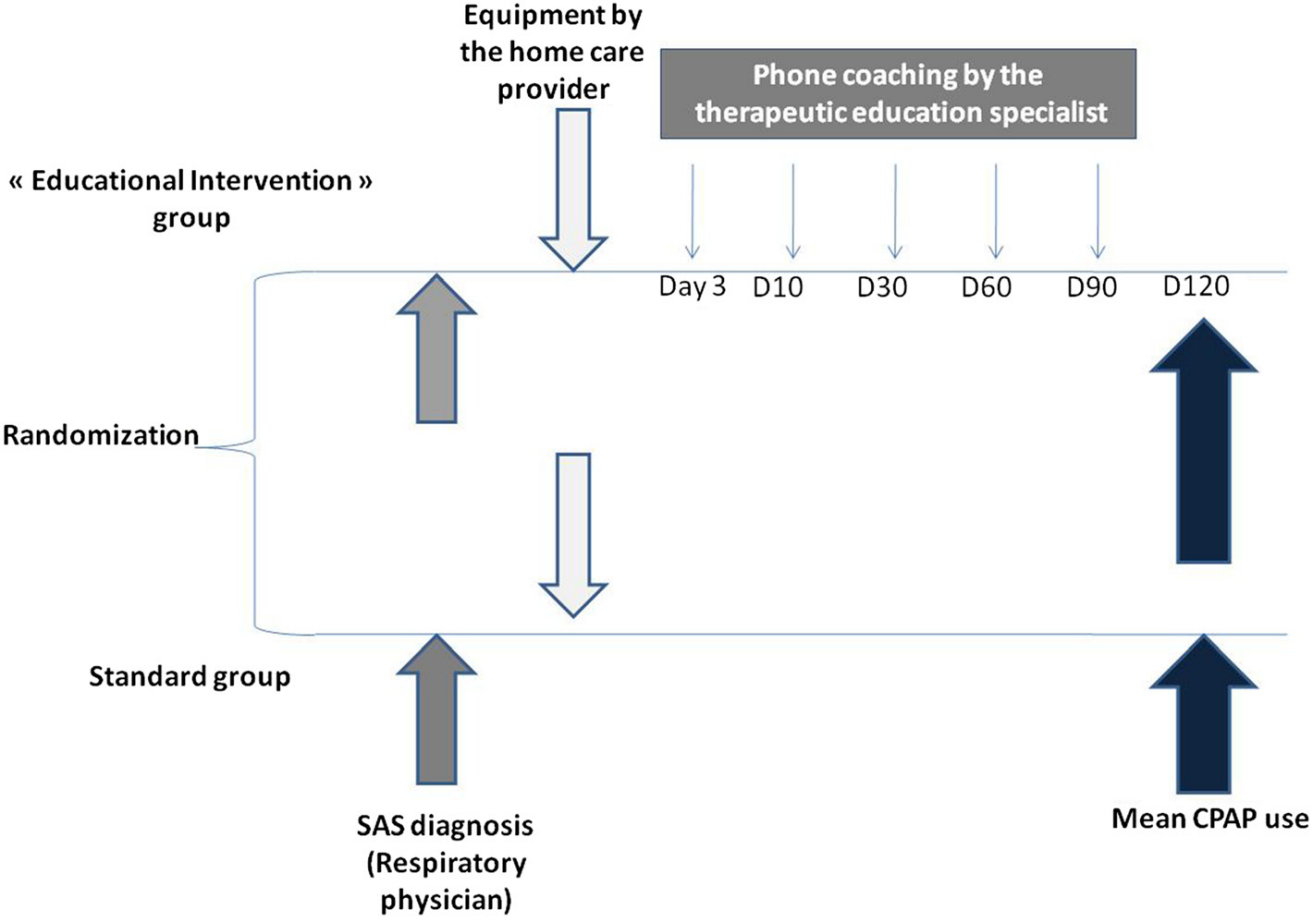
Design of study

We conducted a multicenter, prospective, randomized, controlled, parallel group trial of standard support versus phone coaching for newly diagnosed SAHS patients starting CPAP therapy (cf. Fig. 1). This study has been recorded by AFSSAPS with the RCB number: 2009-A01127-50 and was received and approved on January 2010 by the Ethics Committee in France (French consultative committee for the protection of persons: CPP du Sud-Ouest et Outre-Mer).

### Subjects

Patients for clinical polysomnographic evaluation were recruited from April 2010 to March 2012. Those who were subsequently diagnosed with SAHS and prescribed CPAP were included in the study. Prescription of CPAP treatment was based on French recommendations [24] : daytime sleepiness and more than three of the following criteria: snoring, morning headaches, reduced attention, nocturia, AHT, decreased libido, associated with Apnea Hypopnea Index (AHI) ≥30/h. If AHI was below 30/h a polysomnography was performed and more than 10 arousals/h were required. Eligibility criteria for this study included the ability to understand and speak fluent French and to be able to complete the study questionnaires. Those who were <18 years old, under guardianship, who had previously used CPAP, had a psychiatric illness or were participating in another clinical trial were excluded from participation. All participants signed an informed consent approved by the Human Studies Committee in France (Sud-Ouest et Outre-Mer).

The patient population was then randomized into two groups, one that received standard CPAP support only and the other standard support completed by a coaching session.

Randomization of patients was performed 1/1 ratio at each individual center, by an automatic computer system.

### Procedure

#### Standard support

All patients in this study underwent this procedure, which is the regular procedure in France. In short, the patient received information by their physician, about modalities and usefulness of CPAP treatment. In the week following this information, a technician from the home care provider (SADIR based in France) brought CPAP equipment to the home, re-explained the device function and checked the mask adaptation to the patient. The follow-up of the patient by the home care provider consisted of one visit at home the first month to check the mask’s tolerance and the functioning of the machine. An other visit was performed after 4 months to assess CPAP parameters (length of use, mask leaks, and residual AHI). Sleep physician checked the compliance and efficiency of CPAP treatment once the first month, then at 3 and 6 months. The compliance was then assessed by patient questioning and by looking at the data registered by the machine. After this period, the medical follow up was performed once a year.

#### Coached group

In the coached group (CG), patients received standard support completed by 5 sessions (day 3, 10, 30, 60, 90 with equipment at home) of telephone-based counselling session by competent staff. Sessions were performed by a qualified person in education, qualifies by a university degree (Paul Sabatier University, Toulouse, France). The dates of phone calls were planned with the patient availabilities.

The objective of the first session was to assess the patient’s knowledge about the disease, device and health consequences. The importance of good adherence was emphasized, encouraging the patients to use the CPAP device throughout sleep every day. Objectives of the other educational sessions were first to identify disadvantages or obstacles to follow CPAP treatment and then focus on the benefits linked to use of CPAP. A particular effort was made to discuss misconceptions about sleep apnea and barriers to use, concerns fears and beliefs, as well as the perceptions of their partners and family, in order to increase patients’ positive expectations regarding CPAP benefits. The qualified person in education could also refer any problems in links with SAHS encountered by the patient to the technician, psychologist or dietician (employed by the home care provider). They can respectively help the patient with CPAP technical advice, mentally blocked with CPAP or diet counseling. The average length of each phone call was approximately 15 to 20 min.

#### Assessments and outcome measurements

Demographic data, medical history, and symptoms linked to SAHS were obtained by the physician before polysomnographic evaluation. Subjective daytime sleepiness was measured using the Epworth Sleepiness Score (ESS) [25]. AHI was evaluated by polygraphy completed by polysomnography to determine number of arousals if AHI <30/h. In France, the condition for reimbursement by the national health system was a length of use of 3 or more hours per night over a 5 month period. This is why we chose a cut-off of more than 3 h per night to define good CPAP compliance.

The primary outcome was the proportion of patients using CPAP for more than 3 h per night for 4 months; the secondary outcome was mean hours of CPAP usage in the 2 groups.

The number of phone calls received by the patient was noted and constituted coached subgroups of patients. Mean hours of CPAP usage was also analyzed in these patient subgroups.

### Statistical analysis

Analysis was by intention to treat. Outcome data from patients randomized in the coached group were analyzed even if they dropped out of the study or refused phone calls. The sample characteristics were evaluated using summary statistics with values given as mean (standard deviation) or proportions as appropriate.

Outcomes between groups were compared with independent samples t-tests and the χ^2^ test for the difference in proportions. The number of calls for the coached group were indicated and used for subgroups analysis.

Based on the primary analysis, it was estimated that 352 patients would be required to complete the study to detect a difference of 15 % between two randomized groups, with a 2-tailed significant level of 0.05, and power 0.80. The recruitment target was 380 patients to account for loss to follow up. Stata SE version 9.2 was used for this analysis.

## Results

### Characteristics of patients

**Fig. 2:**
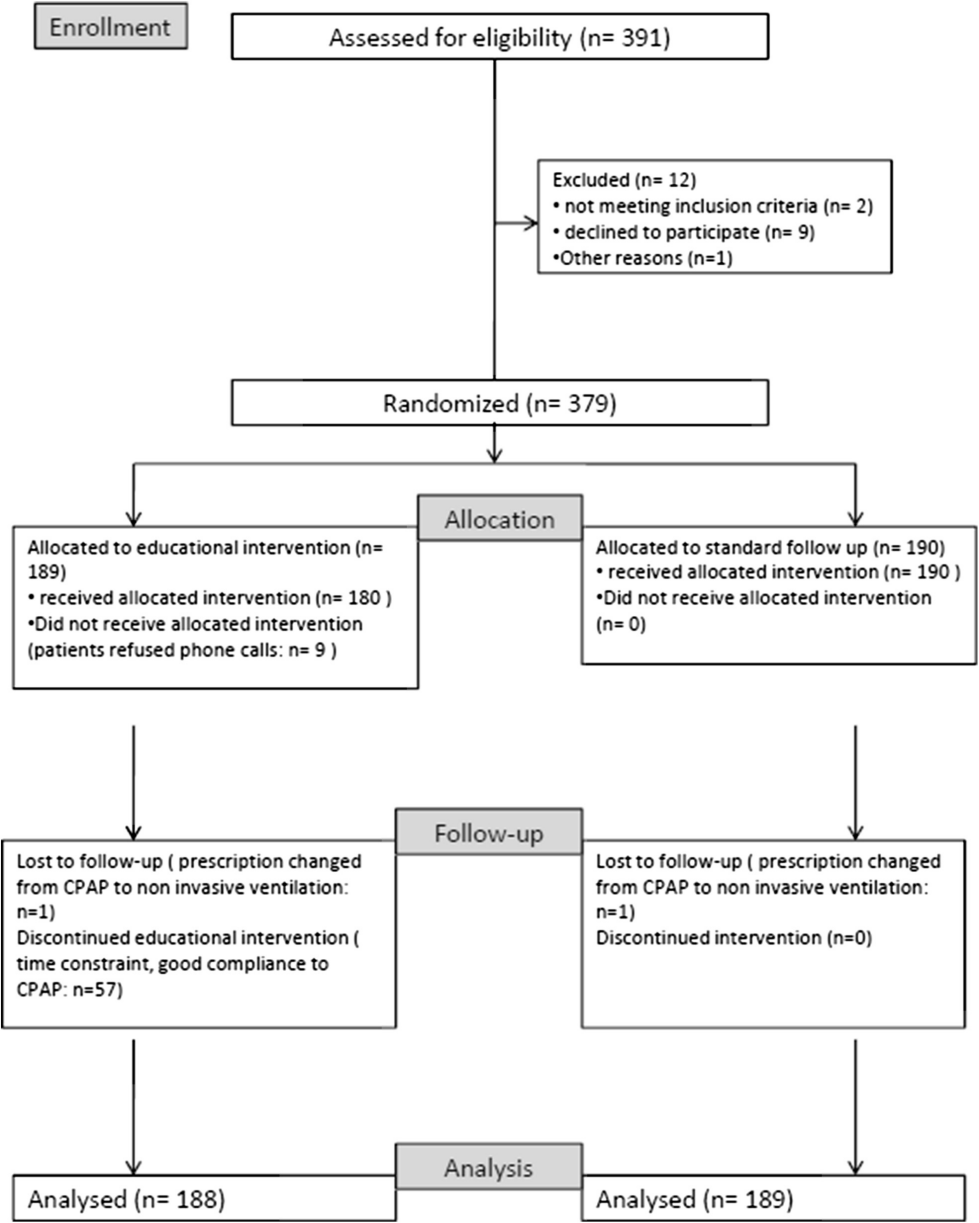
CONSORT flow diagram

Three hundred and seventy-nine patients fulfilled the inclusion criteria and were randomized. All patients had an education level superior to elementary school. Analyzed data are presented on 377 patients (cf. Fig. 2). Patients’ characteristics of the 2 groups are presented in Table 1. Mean age, sex ratio, AHI, ESS were not statistically different among the two randomized groups. A majority of the patients were men, the mean AHI was 42/h. The most common clinical features were a history of snoring (82 %), excessive daytime sleepiness (69 %) and witnessed apneas (30 %). Most patients had associated co morbidity, such as AHT (45 %) or diabetes (20 %).

**Table 1:** Patients’ characteristics at baseline

Characteristics	EI group (*n* = 188)	Standard group (*n* = 189)	*p* value
Age, years, mean+/− SD	58.9+/− 13.7	60.8 +/− 12.6	NS
Male subjects, n (%)	74.6	69.5	NS
BMI kg/m^2^, mean+/− SD	32.8 +/− 6.3	33.1 +/− 7.5	NS
ESS (/24), mean+/− SD	11.7 +/− 4	11.4 +/− 4	NS
% of patients with ESS >10	76.8 %	71.4 %	NS
AHI, events/h, mean+/− SD	41.5 +/− 18.3	42.9 +/− 18.1	NS
% of patients with AHI >30/h	78.4 %	79.4 %	NS

### CPAP use after 4 months between the 2 groups

**Fig. 3:**
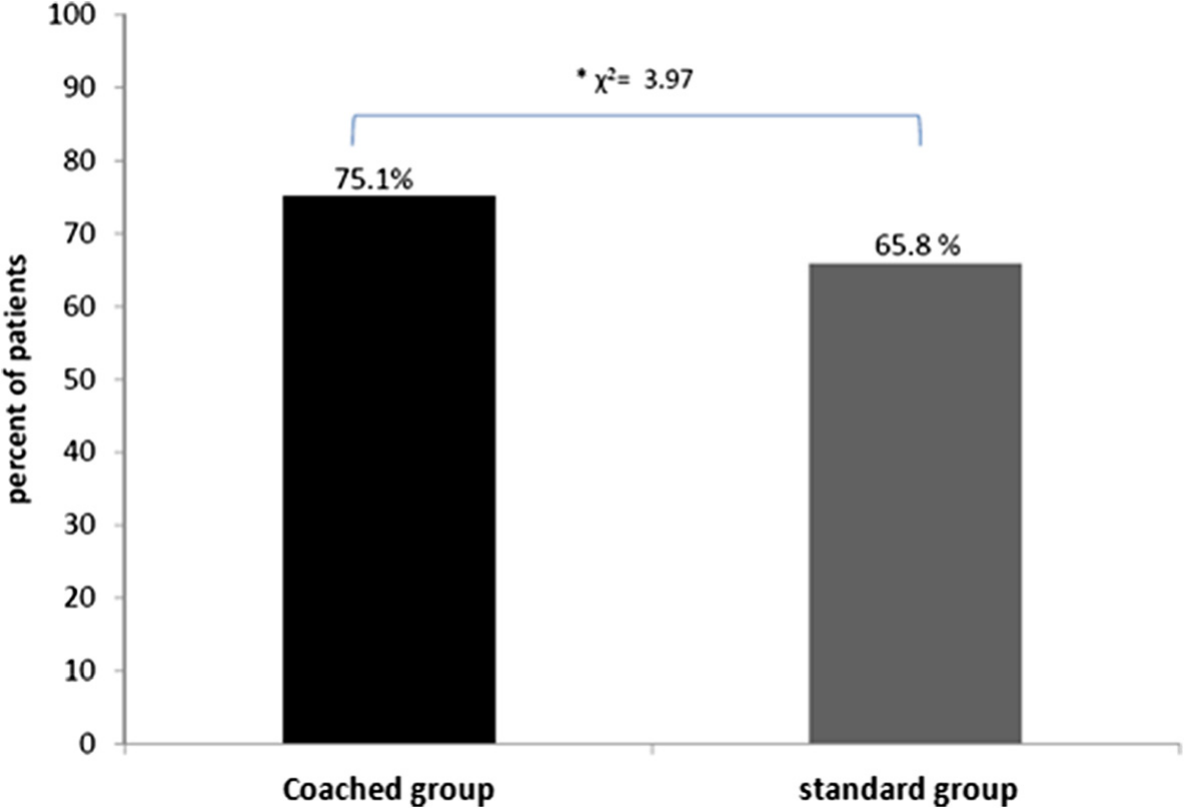
Proportion of patients used CPAP more than 3 h per night for 4 months in coached group (black line) versus standard group (grey line)

Figure 3 shows results for the primary outcome. The percentage of patients using CPAP more than 3 h per night for 4 months was 65 % for the standard support group and 75 % for the coached group. This difference reached statistical significance (χ^2^ = 3.97).

**Fig. 4:**
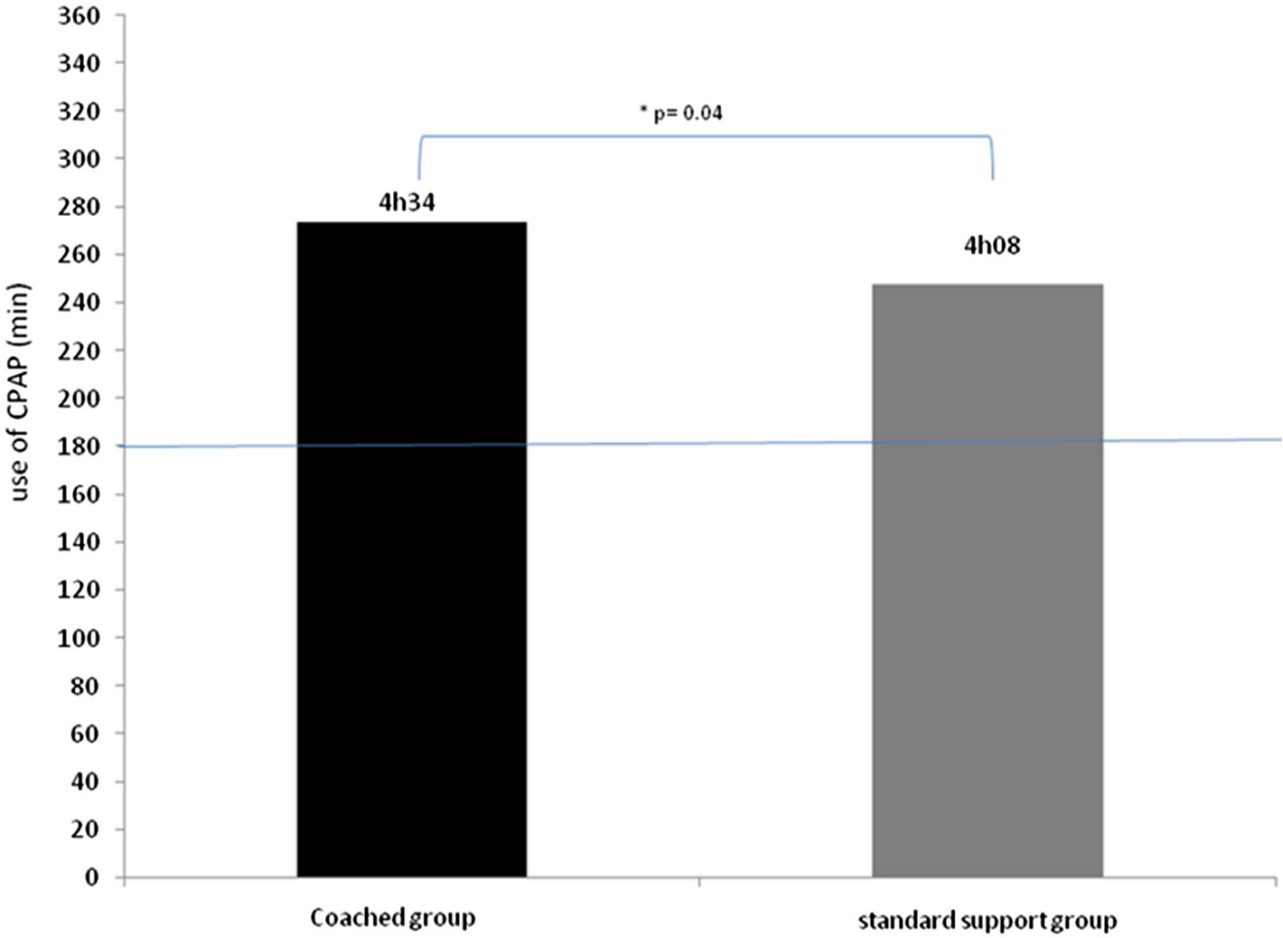
Mean CPAP use (in minutes) for 4 months for patients in coached group (black line) versus standard group (grey line). *p <0.05

For the secondary outcome, the mean CPAP usage was increased for CG versus standard group. A benefit of 26 min was observed (4 h34+/−2 h17 and 4 h08+/−2 h25 respectively) (Fig. 4). This difference was statistically significant (*p* = 0.04).

**Fig. 5:**
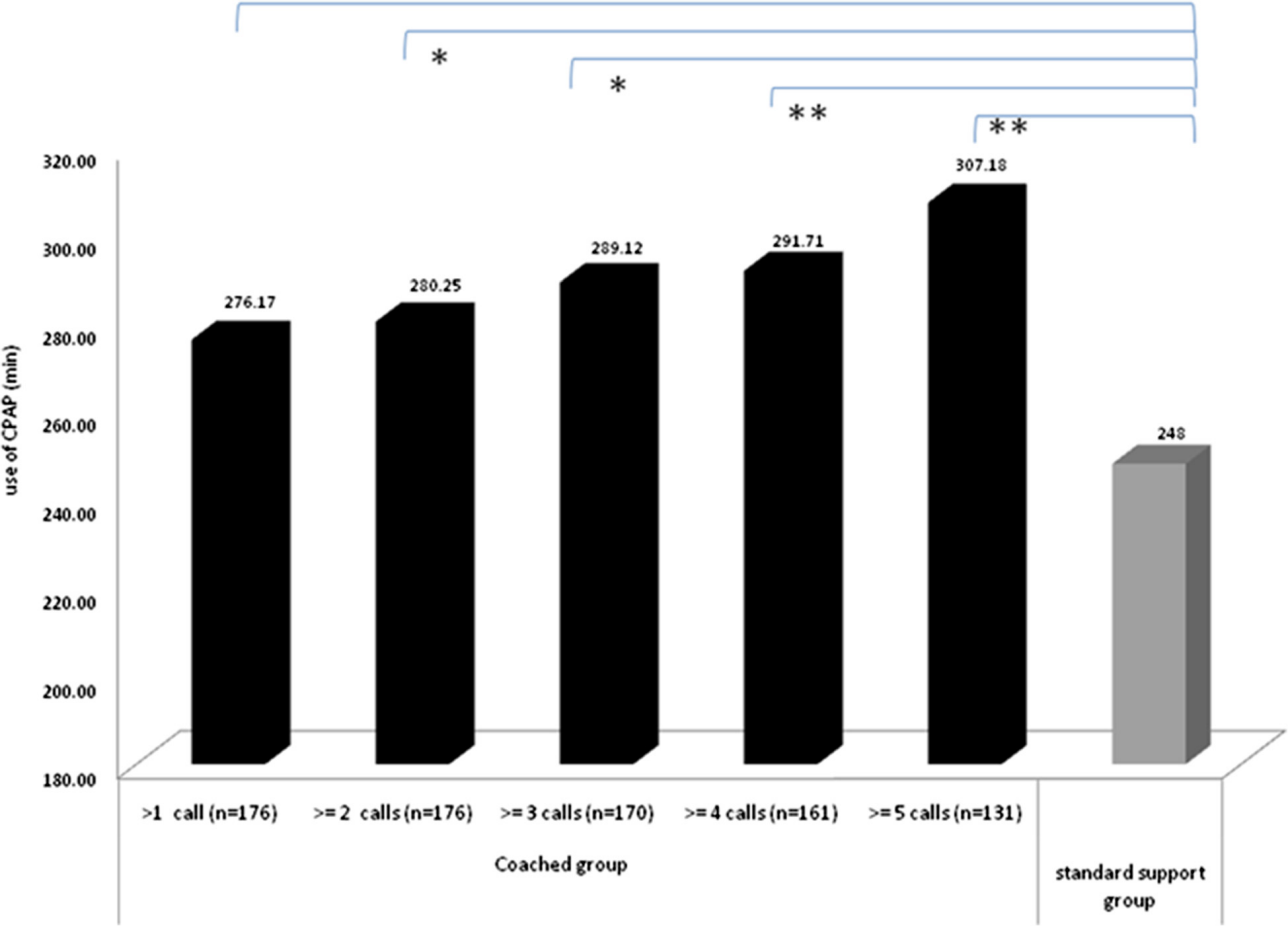
Mean CPAP use (in minutes) for 4 months for subgroups of patient in coached group (black lines) or in standard group (grey line). *p <0.05; **p <0.005

Not all patients with coaching received the 5 phone calls as prescribed in the procedure due to business activity, holidays or patient requests to stop the phone calls. 85 % of patients in CG received at least 4 phone calls and 70 % received 5 phone calls. In Fig. 5, we observed a significant and gradual link between patient phone calls received and mean hours of CPAP use. In the coached subgroups that have received at least 4 phone calls, the use of CPAP was the highest compared to standard group. Patients who respected the completed procedure of 5 phone calls used their CPAP average one hour more than the standard group with a mean of 5 h07 per night (*p* = 0.00015).

## Discussion

In this multisite randomized study, we examined the impact of a phone coaching procedure on CPAP compliance by newly equipped SAHS patients.

This study shows that SAHS patients who benefit from phone coaching are statistically more compliant to CPAP than a standard support group. The number of patients that wore CPAP for more than 3 h was significantly higher in the coaching group. The difference in the use of CPAP between the 2 groups was 28 min and reached one hour in the subgroup of patients who completed the entire procedure (5 phone calls). Several studies demonstrated a good correlation between the length of CPAP use and clinical benefits [7, 13, 26]. Moreover, in France, a minimal length of 3 h CPAP use is mandatory for reimbursement by the national health system [13].

Several educational programs have been proved to increase CPAP compliance. A Cochrane Meta-analysis of 17 randomized trials has confirmed that educational programs improved adherence to CPAP. An increase of a mean of 59 min in the length of use of the device was observed [27]. However these educational programs were usually not made available at home.

The content and length were highly variable and sometimes included participation in a group approach [18, 21, 28]. Finally, the number of patients included in most of the studies was low. Recently, a large (*n* = 3100) and randomized study was published by Bouloukaki I. et al. in a Greek test group [28]. CPAP adherence was compared between standard or intensive use. An increase in CPAP use of 1 h42 was observed in the intensive group after 2 years. This intensive intervention was associated with an increased cost of 30 % versus the standard use. Moreover the application of such intensive procedures in other health systems is questionable.

Our procedure was exclusively practised by phone. Smith et al. observed an improvement with a home educational program combining music and habit-forming use [22]. However, their observed benefits were not maintained at 3 and 6 months.

Our program was focused on exploring the knowledge of the disease by the patient and on motivation reinforcement by highlighting benefits of the CPAP. Two meta-analyses [29, 30] examined factors that improve compliance in SAHS patients and confirmed that the understanding of the disease is one of the key factors for treatment success. Interestingly, Trupp et al. [20] tested negative framing messages emphasizing negative consequences that may occur if CPAP was not worn and found this strategy superior to delivering positive messages about benefit of regular CPAP use. In our study only positive messages were delivered to the patient.

The present study also shows that the mean duration of CPAP use increased with the number of phone calls received by the patient. The higher length of CPAP wear was observed in the patient subgroup that had 5 phone calls as planned by the protocol.

However the difference between the coaching group and the control was significant for all the patients that had at least 2 phone calls. Acceptance of the procedure was excellent, with almost 70 % of the patients accepting the 5 phone calls. Only 12 patients (6 %) had one or zero phone calls. In a previous published study designed to test the feasibility of the phone coaching procedure 86 % of patients had all 5 phone calls [23]. The main reason for discontinuing the procedure was time constraints. These good results might be due to the flexibility of the procedure. Patients kept their autonomy; they could refuse phone calls and were able to decide the phone call schedule. Moreover for the patient, the constraint is minor (5 phone calls in 3 months) and probably balanced by the benefits obtained with the treatment.

## Conclusion

In conclusion, we have demonstrated the efficiency of a simple phone coaching procedure based on knowledge of the disease and reinforcement messages about treatment benefits to improve CPAP adherence in SAHS patients. Moreover, the procedure was well accepted by patients. As a main consequence phone coaching is now systematically performed by our structure in newly equipped patients.

## Abbreviations

AFSSAPS: Agence Française de Sécurité Sanitaire des Produits de Santé
AHI: Apnea hypopnea index
BMI: Body mass index
CPAP: Continuous positive airway pressure
CG: Coaching intervention group
ESS: Epworth sleepiness scale
SAHS: Sleep apnea hypopnea syndrome
RCB: Recherche et collections biologiques
SD: Standard deviation
